# The impacts of Chinese drug volume-based procurement policy on the use of policy-related antibiotic drugs in Shenzhen, 2018–2019: an interrupted time-series analysis

**DOI:** 10.1186/s12913-021-06698-5

**Published:** 2021-07-08

**Authors:** Ying Yang, Lei Chen, Xinfeng Ke, Zongfu Mao, Bo Zheng

**Affiliations:** 1 School of Health Sciences, Wuhan University, 115# Donghu Road, 430071 Wuhan, China; 2 Global Health Institute, Wuhan University, 115# Donghu Road, 430071 Wuhan, China; 3 Institute of Clinical Pharmacology, Peking University First Hospital, No. 8 Xishiku Street, Xicheng District, 100034 Beijing, China; 4 Department of Pharmacy, Peking University First Hospital, No. 8 Xishiku Street, Xicheng District, 100034 Beijing, China

**Keywords:** National Centralized Drug Procurement (NCDP) policy, "4 + 7", volume-based procurement, antibiotic use, China

## Abstract

**Background:**

In 2019, Chinese government implemented volume-based procurement of 25 drugs in 4 municipalities and 7 sub-provincial cities, i.e. “4 + 7” policy. Competitive bidding was conducted by the government based on the annual agreed procurement volume submitted by each public medical institution in pilot cities. Pilot cities were required to implement bid winning results in March 2019 and the use volume of bid winning products was examined to ensure the completion of agreed procurement volume. In the policy, an oral antibiotic (cefuroxime) was included. Given the current condition of the irrational use of antibiotics in China, this study aims to evaluate the impact of “4 + 7” policy on the use of policy-related antibiotics.

**Methods:**

This study used drug purchase data from the Centralized Drug Procurement Survey in Shenzhen 2019, covering 24 months from January 2018 to December 2019. Oral antibiotic drugs related to “4 + 7” policy were selected as study samples, including cefuroxime and 12 antibiotic drugs that have an alternative relationship with cefuroxime in clinical use. Purchase volume and expenditures were selected as outcome variables, and were measured using Defined Daily Doses (DDDs) and Chinese yuan, respectively. Segmented linear regression analysis with interrupted time series was adopted to examine the effect of “4 + 7” policy.

**Results:**

After the implementation of “4 + 7” policy, the overall volume of cefuroxime and its alternative drugs increased from 9.47 million DDDs to 13.42 million DDDs, with an increase of 41.8 %. The results of segmented linear regression showed that the volume of cefuroxime significantly increased 161.16 thousand DDDs after “4 + 7” policy (95 % *CI*: 59.43 to 262.90, *p*-value = 0.004). The volume of alternative drugs significantly increased 273.65 thousand DDDs (95 % *CI*: 90.17 to 457.12, *p*-value = 0.006). The overall “4 + 7” policy-related antibiotics significantly increased 436.31 thousand DDDs (95 % *CI*: 190.81 to 681.81, *p*-value = 0.001) after “4 + 7” policy.

**Conclusions:**

This study provides evidence that the implementation of “4 + 7” volume-based procurement policy was associated with significant increases in the volume of policy-related antibiotic drugs. The increase in antibiotic use after the policy needs special attention and vigilance.

**Supplementary Information:**

The online version contains supplementary material available at 10.1186/s12913-021-06698-5.

## Background

The overuse and misuse of antibiotics stimulated the more rapid emergence of antibiotic resistant bacteria and antibiotic resistant genes, reducing their therapeutic potential against human and animal pathogens [1, 2]. The rising of antibiotic resistant levels, in combination with a lack of new effective antibiotics, increases the morbidity and mortality of infectious diseases, as well as driving inflation related to healthcare costs [3]. World Health Organization (WHO) characterized antimicrobial resistance as a global public health crisis that must be managed with the utmost urgency [4].

China is one of the world’s largest producers and consumers of antibiotics [5]. A nationwide study involving 36 antibiotics reported that 92,700 tonnes of antibiotics were consumed in China, and the DID (defined daily doses for 1000 inhabitants per day) of these 36 antibiotics in China exceed 6 times the UK, Canada, and Europe [6]. Globally, 76 % of the overall increase in antibiotic consumption between 2000 and 2010 was attributable to BRICS countries (Brazil, Russia, India, China, and South Africa) [7]. In BRICS countries, up to 57 % of the increase of antibiotic use in the hospital sector was attributable to China [7]. Yin et al. [8] systematically reviewed the condition of antibiotic utilization in China by using the data of 556,435 outpatient encounters, and reported an overall percentage of 50.3 % for outpatients prescribed antibiotics, of which, 74.0 % were prescribed one antibiotic, 23.3 % were prescribed two antibiotics and 2.0 % were prescribed three or more antibiotics. A national survey showed that 52.9 % of the patients visiting primary care institutions in China were prescribed antibiotics, but only 39.4 % of those who received antibiotics needed them based on their clinical condition [9]. Generally, China has a high prescription use of antibiotics for both inpatients and outpatients [10]. As a result of antibiotic misuse, China has the highest level of antibiotic resistance and the most rapid growth of antibiotic resistance globally [11, 12]. Besides, the direct cost associated with the overuse of antibiotics in China is estimated to be around 2.91 to 13.93 billion yuan ($0.42 to 2.02 billion USD) per year [13]. Therefore, the overuse of antibiotics is an important issue China needs to be vigilant about.

In January 2019, the General Office of the State Council of the People’s Republic of China (PRC) issued the National Centralized Drug Procurement (NCDP) policy, aiming at cutting drug prices and reducing the medication burden of patients [14]. 4 municipalities (Beijing, Tianjin, Shanghai, and Chongqing) and 7 sub-provincial cities (Shenyang, Dalian, Xiamen, Guangzhou, Shenzhen, Chengdu, and Xi’an) in mainland China were selected as pilot cities in the first round of NCDP pilot, thus, this pilot is also known as “4 + 7” policy. The highlight of the “4 + 7” policy lies in the implementation of “volume-based procurement”. Each public medical institution (public hospitals and government-run primary healthcare institutions) in the pilot cities was required to submit the agreed procurement volume for each of 25 drugs to the National Healthcare Security Administration (NHSA) of the PRC. The agreed purchase volume is the expected annual purchase volume of a drug (by generic name) estimated and submitted by the medical institution with reference to the use volume of this drug in the previous year. NHSA of the PRC organized competitive bidding and price negotiation on behalf of pilot cities based on the overall annual agreed procurement volume of 11 pilot cities. Pharmaceutical manufacturers hold original branded drugs that beyond patent protection period and generic drugs that passed the consistency evaluation of quality and efficacy for generic drugs in China are eligible to participate in the bidding. The pharmaceutical manufacturer with the lowest bid price in each drug won the bid. On December 17, 2018, the bid winning results were announced, and the average price reduction of 25 bid winning products was 52 % [15]. 11 pilot cities were required to start implementing the NHSA’s bid winning results in March 2019. The purchases of all the bid winning products were carried out on the provincial drug bidding and procurement platform. Besides, the use volume of each bid winning product in each public medical institution in pilot cities was examined by NHSA to ensure the completion of agreed procurement volume.

In the “4 + 7” policy, most of the 25 drugs are chronic diseases medication for the treat of hypertension, hyperlipidemia, and diabetes [15]. It is worth noting that, a second-generation cephalosporin (i.e. cefuroxime) was included in the 25 drugs, which belongs to Watch group antibiotic according to the 2019 WHO AWaRe classification [16]. According to the bidding results, cefuroxime axetil tablets (250 mg*12 tablets/box) manufactured by Chengdu Brilliant Pharmaceutical Co. Ltd won the bidding at a price of 6.16 yuan/box, which dropped 52.5 % when compared with the average price in the past three years.

Previous studies found that, after the implementation of NCDP policy, the use volume and costs of the drugs that have an alternative relationship with the bid winning product significantly increased [17, 18], which has weakened the cost-saving effect of the policy to a certain extent. The increasing use of alternative drugs, on the one hand, might by related to physicians’ attempts to obtain benefits by prescribing non-centralized purchased drug prescriptions [19]; on the other hand, might be related to the distrust of physicians or patients in the quality of the bid-winning products [20]. It is worth noting that, unlike chronic disease medications, the overuse of antibiotics would accelerate the antimicrobial resistance (AMR), which is recognized as one of the greatest threats to human health worldwide [21, 22]. Therefore, we proposed that the overall consumption of policy-related antibiotics may increase after the implementation policy, that is, the overuse of antibiotics might be exacerbated under “4 + 7” policy. Giving the shortage of qualitative evidence for this issue, we conducted this exploratory study to quantitatively evaluate the effect of “4 + 7” policy on the use of policy-related antibiotic drugs.

## Methods

### Study sites and data sources

In this study, the research site is one of the “4 + 7” pilot cities – Shenzhen. Shenzhen is a megacity in South China, and it forms part of the Pearl River Delta megalopolis. Shenzhen consists of 11 districts and 74 subdistricts, with a total administrative area of 1997.47 km^2^ and a total population of 13.44 million in 2019 [23]. By the end of 2019, Shenzhen has 4,513 medical institutions, of which 114 are hospitals. In Shenzhen, the overall clinical visits are 108,298.7 million in 2019, the drug costs per time per patient in outpatient and inpatient are 84.03 CNY and 304.52 CNY, respectively [24].

This study used data from Centralized Drug Procurement Survey in Shenzhen 2019 (CDPS-SZ 2019). In China, the CDPS-SZ 2019 was organized and conducted by the Global Health Institute of Wuhan University between December 2019 and January 2020. In the survey, monthly drug purchase order data of each included medical institution (hospital and community healthcare center) between 2017 and 2019 were collected. CDPS-SZ 2019 covered all public medical institutions (public hospitals and government-run community healthcare centers) in Shenzhen, as well as some medical institutions in other cities (Dongguan, Zhaoqing, and Harbin). In the CDPS-SZ 2019 database, each purchase order record included purchase date, generic name, dosage form, specification, pharmaceutical manufacturer, price per unit, purchase volume, purchase expenditures, etc. A general database containing 963,127 monthly aggregated purchase order records was established, involving 1079 drug varieties (by generic name), 346 medical institutions, 857 pharmaceutical manufacturers. The total purchase expenditures reached 20.87 billion RMB.

### Samples

**Fig. 1:**
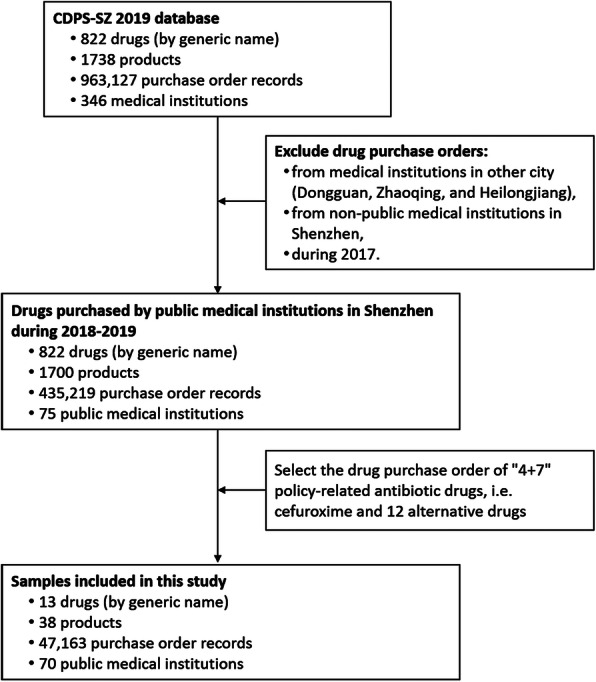
Flow chart of samples screening. Note: CDPS-SZ 2019, Centralized Drug Procurement Survey in Shenzhen 2019

This study aims to examine the effect of “4 + 7” policy on the utilization of policy-related oral antibiotic agents. Thus, we included samples with the following criteria: (a) the drug scope was “4 + 7” policy-related antibiotic drugs, including cefuroxime and the alternative antibiotic drugs. The alternative drug refers to antibiotic drugs that have an alternative relationship with cefuroxime in clinical use, which was determined based on two channels in this study. The first channel is the *Monitoring Plan Work of National Centralized Drug Procurement and Use* issued by the NHSA of the PRC [25]. The document provided a list of alternative drugs for each of the 25 centralized purchased drug, of which 7 oral cephalosporins were identified as the alternative drugs of cefuroxime. The second channel is the recommendation of infection specialists based on the guideline for the clinical use of antibiotic drugs [26] and their clinical experiences. A list of the oral alternative antibiotic drugs that have a strong alternative relationship with cefuroxime in clinical use was provided, involving the category of oral penicillin and oral fluoroquinolones. After removing the repeating items, 12 alternative antibiotic drugs that had been used in the public medical institutions in Shenzhen were included (supplementary Table 1). Cefuroxime was divided into winning products and non-winning products according to the “4 + 7” bid winning results [15]. Winning products refers to cefuroxime produced by Chengdu Brilliant Pharmaceutical Co. Ltd, and non-winning products refers to cefuroxime produced by other pharmaceutical manufacturers. (b) the time period is between January 2018 and December 2019. (c) the medical institution covered all the public medical institutions in Shenzhen. Finally, 9577 purchase order records of 13 drugs (by generic name) were extracted, involving 70 public medical institutions, 30 pharmaceutical manufacturers. Figure 1 presents the flow chart of samples screening.

### Outcome measures

This study assessed the effect of “4 + 7” policy on both volume and expenditures of antibacterial agents. Expenditure data was reported in Chinese yuan, i.e. RMB. Volume was measured using Defined Daily Dose (DDD), a measurement developed by WHO to compare drug consumptions. DDD refers to the average maintenance dose per day for a drug used for its main indication in adults. In this study, DDD value of each medication is determined according to the *Guidelines for ATC classification and DDD assignment 2020* [27]. DDD equivalence per package (DPP) of medicines was calculated in DDD units (DPP = unit strength × pack size/DDD) [28]. The total volume for each group of procured medicines (DDDs) was estimated as the summed DPPs of all-inclusive products.

$$DDDs=\sum_{i=1}^n({DPP}_i\times N_i)$$

Where, *N*_*i*_ represents the number of packages of a certain product (*i*) delivered to the medical institutions.

### Statistical analysis

Descriptive statistics were used. We first described the volume and expenditures of included medications in the same period before (April to December 2018) and after (April to December 2019) the implementation of “4 + 7” policy. Then, we created graphical displays of the monthly procurement volume and expenditures of each study medication in order to observe and describe patterns over time from January 2018 to December 2019.

Interrupted time-series (ITS) analysis was applied to assess the effect of “4 + 7” policy on purchase volume and expenditures of Cefuroxime and related medications. ITS is a commonly used approach for evaluating changes in longitudinal series following a quasi-experimental intervention occurring at a fixed point in time, such as the date of implementing “4 + 7” policy, i.e. 1 April 2019 in Shenzhen. We constructed interrupted time series using drug procurement data in Shenzhen from January 2018 to December 2019. The time unit was set to 1 month and the intervention time point was set to April 2019, making 24 time points available for analysis, including 15 points before the intervention and 9 points thereafter. To estimate the effect of the intervention on the outcome variables, the following segmented linear regression model was developed [29]:

$${Y}_{t}={\beta }_{0}+{\beta }_{1}\times {time}_{t}+{\beta }_{2}\times {intervention}_{t}+{\beta }_{3}\times time after {intervention}_{t}+{\beta }_{4}\times cold+{\epsilon }_{t}$$

Where, *Y*_*t*_ is the independent outcome variable (volume or expenditures) in month *t*; *time* is a continuous variable indicating time in months at time *t* from the start of the observation period; *intervention* is an indicator for time *t* occurring before (*intervention* = 0) or after (*intervention* = 1) “4 + 7” policy, which was implemented at month 15 in the series; and *time after intervention* is a continuous variable indicating months passed since the intervention (time prior to the intervention is coded 0). Besides, we set a dummy variable *cold* to control the extreme value of antibiotic use during the Spring Festival holiday, which is “wild data points” in this study [29]. It is when the Spring Festival that is the coldest time of the year in China, and common seasonal illness (cold) prevalence. The possibility of antibiotics overuse rises to treat fever and other associated symptoms [30–32]. The variable *cold* is assigned the value 1 in December and January of each year and 0 otherwise.

In this model, *β*_*0*_ estimates the baseline level of the independent variable at the beginning of the observation period. *β*_*1*_ estimates the linear trend during the pre-intervention period where time *t* is an integer variable indicating the time in months at time *t* from the beginning of the study period. *β*_*2*_ estimates the change in the outcome immediately following the intervention. *β*_*3*_ estimates the change in trend in the outcome measures after the intervention compared with the monthly trend before the intervention. *Β*_*4*_ estimates the “coldest weather” effect. *ε*_*t*_ is an estimate of the random error at time *t*. Durbin-Watson test was performed to test the presence of first-order auto-correlation (a value around 2 indicates no sign of auto-correlation). If auto-correlation is detected, the Prais-Winsten method was applied to estimate the regression. Stata version 16.0 was used to perform the ITS analysis.

## Results

### Descriptive statistics

A total of 13 antibiotic drugs (by generic name) were included in this study, involving 38 antibiotic products from different pharmaceutical manufacturers. The total purchase volume was 29.33 million DDDs, and the total purchase expenditure was 266.83 million RMB. Of those, the volume and expenditures of cefuroxime were 6.39 million DDDs and 12.74 million RMB, respectively.

Table 1 demonstrates the change of volume and expenditures of antibiotic medications included in this study during the same period before (April to December 2018) and after (April to December 2019) the implementation of “4 + 7” policy. Compared with April to December 2018, the purchase volume of cefuroxime during April to December 2019 increased by 92.9 %, and the purchase expenditures decreased by 18.9 %. Of which, the volume and expenditures of winning products increased by 182.9 and 102.4 %, respectively; those of non-winning products decreased by 70.3 and 74.1 %, respectively. From April to December 2019, the volume and expenditures of cefuroxime’s alternative medication increased by 30.2 and 20.1 %, respectively, when compared with those from April to December 2018. Besides, the overall volume of all included antibiotics in this study rose 41.8 % and the expenditures rose 18.8 %.

**Table 1:** Purchase volume and expenditures of included antibacterial agents in April to December 2018 and April to December 2019

Categories	Volume (million DDDs)			Expenditures (million RMB)		
	Apr. to Dec. 2018	Apr. to Dec. 2019	Relative change (%)	Apr. to Dec. 2018	Apr. to Dec. 2019	Relative change (%)
Cefuroxime	1.75	3.38	92.9	5.19	4.21	-18.9
Winning products	1.13	3.20	182.9	1.62	3.29	102.4
Non-winning products	0.62	0.18	-70.3	3.56	0.92	-74.1
Alternatives	7.71	10.04	30.2	87.49	105.89	21.0
Total	9.47	13.42	41.8	92.67	110.09	18.8

### ITS analysis

Changes of winning and non-winning products.

**Fig. 2:**
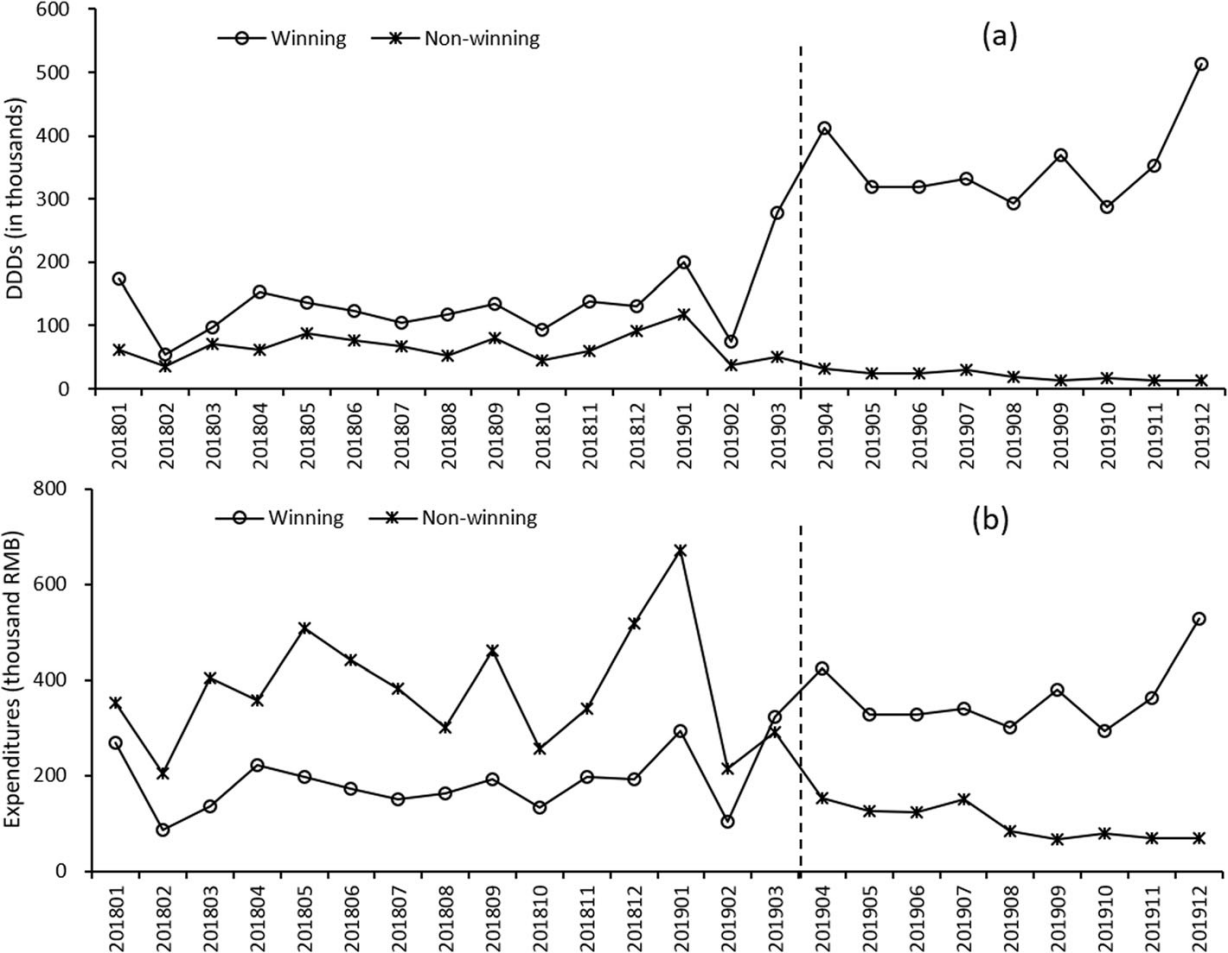
Trends of monthly drug purchase volume and expenditures for winning and non-winning products. (**a**) Volume (thousand DDDs); (**b**) Expenditures (thousand RMB)

The monthly trends of volume and expenditures of winning and non-winning cefuroxime products are displayed in Fig. 2. Table 2 shows the results of the segmented regression analysis for the volume of winning and non-winning products. After the implementation of “4 + 7” policy, winning products significantly increased 185.07 thousand DDDs (95 % *CI*: 80.13 to 290.02, *p*-value = 0.002). The trend of volume decreased by 0.82 thousand DDDs per month but with no statistically significant (*p*-value = 0.920). Non-winning products decreased by 26.35 thousand DDDs (*p*-value = 0.096) and the trend of volume decreased by 4.27 thousand DDDs per month (*p*-value = 0.091), but no significant differences were observed. As for expenditures (supplementary Table 2), winning products significantly increased 158.92 thousand RMB after “4 + 7” policy (95 % CI: 57.34 to 260.50, p-value = 0.004).

**Table 2:** Results of the segmented linear regression models for the volume of winning and non-winning products

	Coefficient	Standard Error	*t*	*p*-value	95 % *CI*	
					Lower	Upper
**Model 1, Winning products**						
Secular trend, *β*_*1*_	3.94	3.28	1.20	0.245	-2.92	10.80
Change in level, *β*_*2*_	185.07	50.14	3.69	0.002	80.13	290.02
Change in trend, *β*_*3*_	-0.82	8.01	-0.10	0.920	-17.58	15.95
Cold, *β*_*4*_	73.25	31.36	2.34	0.031	7.61	138.89
Constant, *β*_*0*_	87.74	30.10	2.92	0.009	24.75	150.73
**Model 2, Non-winning products**						
Secular trend, *β*_*1*_	0.33	0.98	0.33	0.744	-1.73	2.38
Change in level, *β*_*2*_	-26.35	15.03	-1.75	0.096	-57.81	5.11
Change in trend, *β*_*3*_	-4.27	2.40	-1.78	0.091	-9.30	0.75
Cold, *β*_*4*_	23.97	9.41	2.55	0.020	4.27	43.66
Constant, *β*_*0*_	59.08	9.02	6.55	0.000	40.21	77.95

Model 1, *F* = 25.18, *p*-value < 0.001, *R*^*2*^ = 0.841, Adjusted *R*^*2*^ = 0.808; Model 2, *F* = 13.02, *p*-value < 0.001, *R*^*2*^ = 0.733, Adjusted *R*^*2*^ = 0.676

### Changes of cefuroxime and alternative drugs

**Fig. 3:**
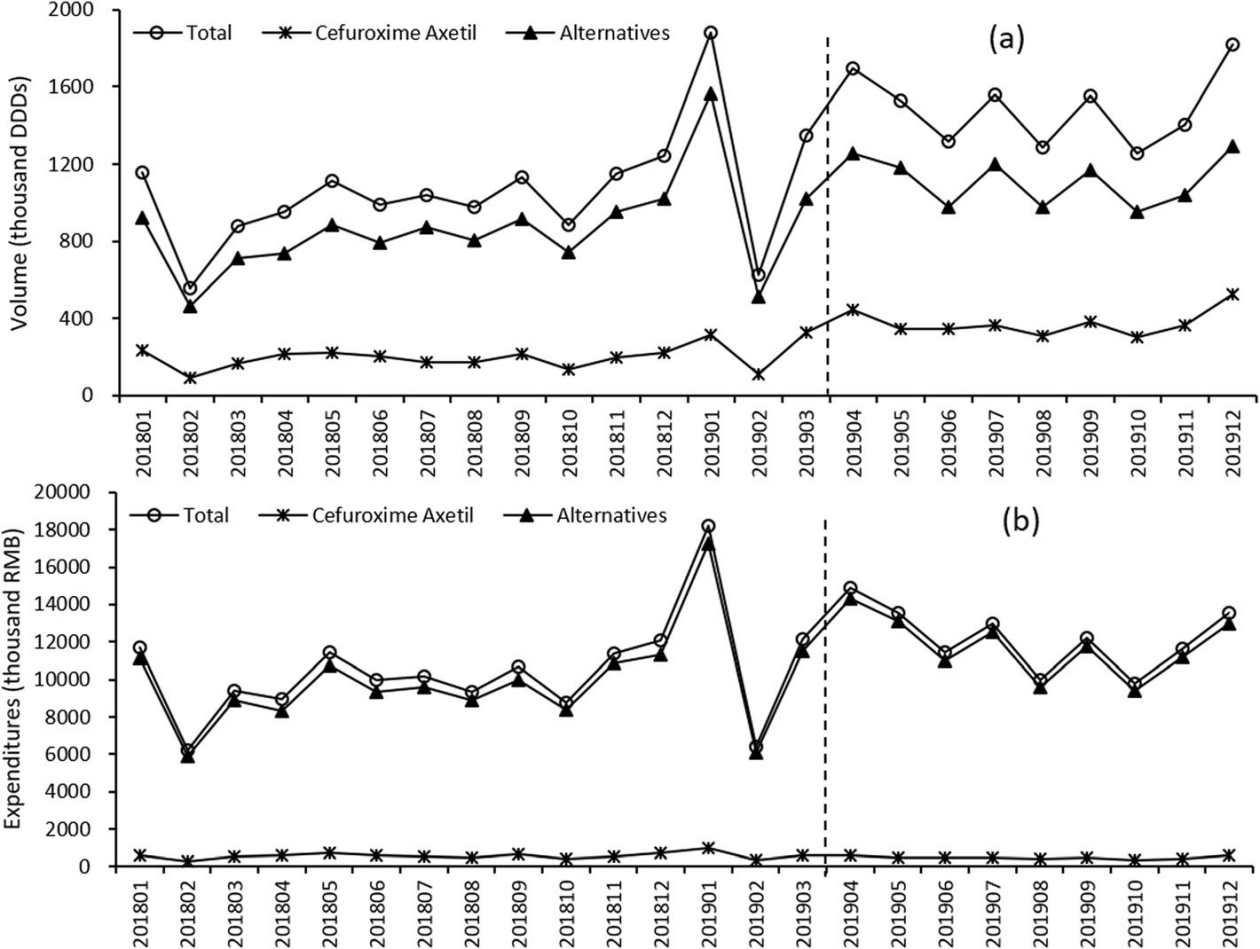
Trends of monthly drug purchase volume and expenditures for Cefuroxime Axetil and its Alternatives. (**a**) Volume (thousand DDDs); (**b**) Expenditures (thousand RMB)

The monthly trends of volume and expenditures of cefuroxime and its alternative drugs are displayed in Fig. 3. Table 3 shows the results of the segmented regression analysis of the volume of cefuroxime and its alternative drugs. After the implementation of “4 + 7” policy, cefuroxime significantly increased 161.16 thousand DDDs (95 % *CI*: 59.43 to 262.90, *p*-value = 0.004). The trend of cefuroxime’s volume decreased by 5.41 thousand DDDs per month but with no statistically significant (*p*-value = 0.480). The alternatives drugs of cefuroxime significantly increased by 273.65 thousand DDDs after “4 + 7” policy (95 % *CI*: 90.17 to 457.12, *p*-value = 0.006). The trend of alternatives drugs’ volume significantly decreased by 47.57 thousand DDDs per month (95 % *CI*: -74.59 to -20.25, *p*-value = 0.002). The total volume of selected antibiotic drugs significantly increased 436.31 thousand DDDs after “4 + 7” policy (95 % *CI*: 190.81 to 681.81, *p*-value = 0.001). The trend of volume significantly decreased by 54.09 thousand DDDs per month (95 % *CI*: 90.31 to 17.88, *p*-value = 0.006).

**Table 3:** Results of the segmented linear regression models for the volume of Cefuroxime and its alternative drugs

	Coefficient	Standard Error	*t*	*p*-value	95 % *CI*	
					Lower	Upper
**Model 1, Cefuroxime**						
Secular trend, *β*_*1*_	4.12	3.09	1.33	0.199	-2.35	10.59
Change in level, *β*_*2*_	161.16	48.61	3.32	0.004	59.43	262.90
Change in trend, *β*_*3*_	-5.41	7.52	-0.72	0.480	-21.16	10.33
Cold, *β*_*4*_	89.67	32.04	2.80	0.011	22.62	156.72
Constant, *β*_*0*_	149.22	28.04	5.32	0.000	90.54	207.89
**Model 2, Alternative drugs**						
Secular trend, *β*_*1*_	20.52	5.42	3.79	0.001	9.18	31.86
Change in level, *β*_*2*_	273.65	87.66	3.12	0.006	90.17	457.12
Change in trend, *β*_*3*_	-47.57	12.91	-3.69	0.002	-74.59	-20.55
Cold, *β*_*4*_	313.94	65.63	4.78	0.000	176.56	451.31
Constant, *β*_*0*_	634.46	47.66	13.31	0.000	534.70	734.22
**Model 3, Total**						
Secular trend, *β*_*1*_	24.70	7.25	3.41	0.003	9.52	39.88
Change in level, *β*_*2*_	436.31	117.29	3.72	0.001	190.81	681.81
Change in trend, *β*_*3*_	-54.09	17.30	-3.13	0.006	-90.31	-17.88
Cold, *β*_*4*_	385.09	87.21	4.42	0.000	202.55	567.63
Constant, *β*_*0*_	786.20	63.89	12.30	0.000	652.47	919.94

Model 1, *F* = 19.63, *p*-value < 0.001, *R*^*2*^ = 0.805, Adjusted *R*^*2*^ = 0.764; Model 2, *F* = 32.63, *p*-value < 0.001, *R*^*2*^ = 0.873, Adjusted *R*^*2*^ = 0.846; Model 3, *F* = 36.12, *p*-value < 0.001, *R*^*2*^ = 0.884, Adjusted *R*^*2*^ = 0.859

In terms of expenditures (supplementary Table 3), the alternatives drugs of cefuroxime significantly increased 3471.66 thousand RMB after “4 + 7” policy (95 % *CI*: 1529.70 to 5413.62, *p*-value = 0.001). The trend of alternatives drugs’ expenditures significantly decreased by 658.52 thousand RMB per month (95 % *CI*: -944.20 to -372.83, *p*-value < 0.001). The total expenditures of selected antibiotic drugs significantly increased 3437.80 thousand RMB after “4 + 7” policy (95 % *CI*: 1324.56 to 5551.05, *p*-value = 0.003). The trend expenditures significantly decreased by 680.44 thousand RMB per month (95 % *CI*: -991.63 to -369.25, *p*-value < 0.001).

## Discussion

In this study, using the drug purchase order data of public medical institutions in Shenzhen between January 2018 and December 2019, we analyzed the impact of “4 + 7” policy on the use of policy-related antibiotic drugs by conducting interruption time series analysis. The present findings might provide references for promoting the rational use of antibiotic drugs, as well as the implementation and adjustment of NCDP policy in the following rounds.

This study found that the purchase volume and expenditures of bid winning cefuroxime products significantly increased in Shenzhen after the implementation of “4 + 7” policy, with an increment of 182.9 % and 102.4 %, respectively. The finding is generally consistent with the results of all 25 winning drugs regarding the total purchase volumes and expenditures [18]. Besides, the volume of cefuroxime significantly increased after the implementation of “4 + 7” policy, with an increase of 92.9 %. The prominent increasing use of bid winning products, on the one hand, might suggest that the medication accessibility of winning products was improved for patients, and the medication demand was greatly released after “4 + 7” policy [33]; on the other hand, might be related to the increasing of medication course or single dose in order to complete the agreed purchased volume of winning product [19].

After the implementation of “4 + 7” policy, the volume and expenditures of non-winning cefuroxime products decreased by 70.3 and 74.1 %, respectively. However, the results of segmented linear regression indicated no statically significances. In terms of the original intention of policy design, the NCDP policy hopes that the bid winning drugs under substantial price cut will replace the non-winning drugs as much as possible, so as to archive the goal of reducing drug costs and relieve the overall drug burden of patients [34, 35]. From this point of view, the original intention of this policy may have not been fully achieved.

In this study, ITS analysis indicated that the volume of alternative antibiotic drugs increased significantly after “4 + 7” policy, with an growth rate of 31.2 %. Besides, the volume of the overall policy-related antibiotic drugs increased after “4 + 7” policy, with an increment of 41.8 %. Significant statistical increasing was observed in the ITS analysis of volume for overall policy-related antibiotic drugs. These findings suggested that “4 + 7” policy might promoted the overuse of policy-related antibiotic drugs, The main reason is the increase in the use of alternative drugs, mainly manifested in the increasing use of alternative antibiotic drugs. Chen et al. [17] and Mao et al. [18] found that the volume and expenditures of the drugs that have an alternative relationship with the bid winning product significantly increased after “4 + 7” policy, which has become a common phenomenon in NCDP policy. This study found that the same “side effects” also exist among antibiotic drugs. Regarding the potential mechanism for the overuse of policy-related antibiotic overuse under “4 + 7” policy, on the one hand, due to the pressure of policy assessment, medical institutions increase the use of bid winning cefuroxime (such as increasing the course of treatment and single dose) to ensure the completion of assessment target; on the other hand, considering the potential interests of physicians in drug selection and the distrust of some physicians on bid winning products, the use of non-centralized purchased drugs (i.e. alternative drugs) increased, which was a common phenomenon in the “4 + 7” policy.

This study also found that the expenditures of alternative antibiotics and the overall policy-related antibiotic drugs significantly increased after the implementation of “4 + 7” policy. It suggested that the cost-saving effect of “4 + 7” policy did not present in the antibacterial drugs. Li et al. [36] found that patients’ average drug costs had not been brought down by the price reduction after in implementation of bidding procurement of antimicrobial drugs in Hubei, China. Liu et al.’s survey [37] on 5 county-level public hospitals in Anhui, China reported that the daily drug costs (DDDc) of antibacterial drugs decreased after the implementation of centralized bidding system, while the volumes and expenditures were still increasing. These findings are generally in line with our results in this study, indicating that price cut alone could not effectively curb the increasing trend of antibiotic drugs whether in volume or in expenditures. More importantly, it is necessary to change doctors’ behaviors regarding the prescription of antibiotics [30], so as to promote the rational use of antibiotics and control the growth of drug expenditures.

Overall, the increase in the volume and expenditures of non-centralized purchased drugs is a common phenomenon for most drug categories under “4 + 7” policy [18, 38]. This is so-called “side effect” and very common in pharmaceutical policies [39, 40], that is, the expenditure of the drugs with price cuts was steady or decease, but the use of drugs without price cuts substantially increased. For antibiotic drugs, their overuse is of great hazard [41]. The overuse of antibiotic drugs would accelerate the antimicrobial resistance, which is recognized as one of the greatest threats to human health worldwide [21, 22]. Thus, we believe that the volume-based procurement for antibiotic drugs need to be more cautious. On the one hand, it is recommended to expand the scope of centralized procurement drugs and include antibiotics that have alternative relationships in clinical use at the same time. On the other hand, it is suggested to further promote the guidance and monitoring of the rational use of antibiotics and standardize the prescription behavior of physicians.

Several potential limitations should be mentioned regarding the present study. Firstly, in terms of evaluating policy effect, the two groups of ITS has more advantages than the single group ITS by setting a control group [42]. However, we failed to set a control group in this study, thus it is difficult to observe and control the potential confounding factors (such as other policies) that may affect the results. Secondly, considering the policy intervention time point and the stability of baseline data, this study only included the data for 24 months. The follow-up periods were short from the date when the policy was implemented with only 9 time points after the intervention, which may be insufficient in trend analysis. Thirdly, the results of this study were based on drug purchase data, rather than drug use data (such as prescriptions). Although there is strong consistency between purchase data and use data under NCDP policy. However, there is still possibility that the two data sources may not exactly match, so there are certain limitations.

## Conclusions

This study provides evidence that the implementation of “4 + 7” volume-based procurement policy was associated with significant increases in the volume and expenditure of cefuroxime and its alternative drugs. Given the increasing antibiotic resistance in China, the rising of antibiotic use after the policy needs special attention and vigilance. For the improvement of NCDP policy, it is suggested to expand the scope of centralized procurement drugs and include antibiotics that have alternative relationships in clinical use at the same time. Besides, further guidance and monitoring of the rational use of antibiotics might make sense.

## Supplementary Information

**Additional file 1.**

## Data Availability

The datasets generated or analysed during the current study are not publicly available due confidentiality policies but are available from the corresponding author on reasonable request.

## Abbreviations

WHO: World Health Organization
BRICS countries: Brazil, Russia, India, China, and South Africa
NCDP: National Centralized Drug Procurement
AMR: antimicrobial resistance
CDPS-SZ: Centralized Drug Procurement Survey in Shenzhen
DDD: Defined Daily Dose
DPP: DDD equivalence per package
ITS: Interrupted time-series
CI: Confidence interval
DDDc: Daily Drug Costs
