# Supplementary material for: The impacts of Chinese drug volume-based procurement policy on the use of policy-related antibiotic drugs in Shenzhen, 2018–2019: an interrupted time-series analysis
Source: BMC Health Serv Res. 2021 Jul 8;21:668. doi: 10.1186/s12913-021-06698-5 (PMC8265121; doi:10.1186/s12913-021-06698-5)
Supplement: Supplementary file 1 — Additional file 1. [file 12913_2021_6698_MOESM1_ESM.docx]

**Supplementary table 1.** List of oral policy-related antibiotic drugs included in this study.

| **Category** | **Generic name** | **Number of products** | **Number of pharmaceutical manufacturers** |
| --- | --- | --- | --- |
| Centralized purchased antibiotic drug | Cefuroxime | 5 | 3 |
| Alternate drugs | Amoxicillin | 2 | 2 |
|  | Cefaclor | 8 | 6 |
|  | Norfloxacin | 1 | 1 |
|  | Cefprozil | 2 | 2 |
|  | Cefixime | 4 | 4 |
|  | Moxifloxacin | 1 | 1 |
|  | Amoxicillin and Clavulanate | 4 | 4 |
|  | Ampicillin | 1 | 1 |
|  | Cephalexin | 2 | 2 |
|  | Cefpodoxime | 2 | 2 |
|  | Cefdinir | 3 | 3 |
|  | Levofloxacin | 3 | 3 |

**Supplementary table 2.** Results of the segmented linear regression models for the expenditures of winning and non-winning products.

|  | Coefficient | Standard Error | *t* | *p*-value | 95% *CI* | |
| --- | --- | --- | --- | --- | --- | --- |
|  |  |  |  |  | Lower | Upper |
| **Model 1, Winning  products** |  |  |  |  |  |  |
| Secular trend, *β_1_* | 3.27 | 3.07 | 1.07 | 0.300 | -3.15 | 9.70 |
| Change in level, *β_2_* | 158.92 | 48.53 | 3.27 | 0.004 | 57.34 | 260.50 |
| Change in trend, *β_3_* | -2.66 | 7.47 | -0.36 | 0.726 | -18.29 | 12.97 |
| Cold, *β_4_* | 94.59 | 32.36 | 2.92 | 0.009 | 26.85 | 162.33 |
| Constant, *β_0_* | 143.04 | 27.78 | 5.15 | 0.000 | 84.90 | 201.19 |
| **Model 2, Non-winning  products** |  |  |  |  |  |  |
| Secular trend, *β_1_* | 1.93 | 5.62 | 0.34 | 0.735 | -9.84 | 13.70 |
| Change in level, *β_2_* | -176.39 | 86.10 | -2.05 | 0.055 | -356.61 | 3.82 |
| Change in trend, *β_3_* | -22.48 | 13.74 | -1.64 | 0.118 | -51.24 | 6.28 |
| Cold, *β_4_* | 136.03 | 53.92 | 2.52 | 0.021 | 23.18 | 248.88 |
| Constant, *β_0_* | 337.51 | 51.63 | 6.54 | 0.000 | 229.45 | 445.57 |

Model 1, *F* = 20.02, *p*-value < 0.001, *R^2^* = 0.808, Adjusted *R^2^* = 0.768; Model 2, *F* = 14.17, *p*-value < 0.001, *R^2^* = 0.749, Adjusted *R^2^* = 0.696.

**Supplementary table 3.** Results of the segmented linear regression models for the expenditures of Cefuroxime Axetil and its alternative agents.

|  | Coefficient | Standard Error | *t* | *p*-value | 95% *CI* | |
| --- | --- | --- | --- | --- | --- | --- |
|  |  |  |  |  | Lower | Upper |
| **Model 1, Cefuroxime** |  |  |  |  |  |  |
| Secular trend, *β_1_* | 4.96 | 5.92 | 0.84 | 0.412 | -7.43 | 17.35 |
| Change in level, *β_2_* | -28.45 | 93.99 | -0.30 | 0.765 | -225.17 | 168.27 |
| Change in trend, *β_3_* | -22.85 | 14.37 | -1.59 | 0.128 | -52.93 | 7.22 |
| Cold, *β_4_* | 221.88 | 63.49 | 3.49 | 0.002 | 88.99 | 354.77 |
| Constant, *β_0_* | 486.16 | 53.39 | 9.11 | 0.000 | 374.42 | 597.90 |
| **Model 2, Alternative drugs** |  |  |  |  |  |  |
| Secular trend, *β_1_* | 168.83 | 57.35 | 2.94 | 0.008 | 48.79 | 288.86 |
| Change in level, *β_2_* | 3471.66 | 927.83 | 3.74 | 0.001 | 1529.70 | 5413.62 |
| Change in trend, *β_3_* | -658.52 | 136.49 | -4.82 | 0.000 | -944.20 | -372.83 |
| Cold, *β_4_* | 3630.32 | 697.27 | 5.21 | 0.000 | 2170.91 | 5089.72 |
| Constant, *β_0_* | 7813.30 | 504.01 | 15.50 | 0.000 | 6758.39 | 8868.22 |
| **Model 3, Total** |  |  |  |  |  |  |
| Secular trend, *β_1_* | 173.62 | 62.41 | 2.78 | 0.012 | 43.00 | 304.24 |
| Change in level, *β_2_* | 3437.80 | 1009.66 | 3.40 | 0.003 | 1324.56 | 5551.05 |
| Change in trend, *β_3_* | -680.44 | 148.68 | -4.58 | 0.000 | -991.63 | -369.25 |
| Cold, *β_4_* | 3839.38 | 755.78 | 5.08 | 0.000 | 2257.51 | 5421.26 |
| Constant, *β_0_* | 8304.29 | 549.01 | 15.13 | 0.000 | 7155.20 | 9453.38 |

Model 1, *F* = 5.04, *p*-value = 0.006, *R^2^* = 0.515, Adjusted *R^2^* = 0.413; Model 2, *F* = 26.85, *p*-value < 0.001, *R^2^* = 0.850, Adjusted *R^2^* = 0.818; Model 3, *F* = 23.22, *p*-value < 0.001, *R^2^* = 0.830, Adjusted *R^2^* = 0.795.
